# Cu-ZnO Embedded in a Polydopamine Shell for the Generation of Antibacterial Surgical Face Masks

**DOI:** 10.3390/molecules29184512

**Published:** 2024-09-23

**Authors:** Nicola d’Alessandro, Francesca Coccia, Luca Agostino Vitali, Giorgia Rastelli, Amedeo Cinosi, Andrea Mascitti, Lucia Tonucci

**Affiliations:** 1 Department of Engineering and Geology, “G. d’Annunzio” University of Chieti-Pescara, Viale Pindaro 42, 65127 Pescara, Italy; nicola.dalessandro@unich.it (N.d.); andrea.mascitti@alumni.unich.it (A.M.); 2 TEMA Research Center, “G. d’Annunzio” University of Chieti-Pescara, 66100 Chieti, Italy; lucia.tonucci@unich.it; 3 UdA-TechLab Research Center, “G. d’Annunzio” University of Chieti-Pescara, 66100 Chieti, Italy; 4 Department of Socio-Economic, Managerial and Statistical Studies, “G. d’Annunzio” University of Chieti-Pescara, Via dei Vestini, 31, 66100 Chieti, Italy; 5 School of Pharmacy, University of Camerino via Gentile III da Varano, 62032 Camerino, Italy; luca.vitali@unicam.it; 6 Department of Neuroscience, Imaging and Clinical Science, “G. d’Annunzio” University of Chieti-Pescara, Via dei Vestini, 31, 66100 Chieti, Italy; giorgia.rastelli@unich.it; 7 G.N.R. s.r.l., Via Torino 7, 28010 Agrate Conturbia, Italy; info@gnr.it

**Keywords:** face masks, antibacterial functionalization, copper oxide, zinc oxide, polydopamine

## Abstract

A new easy protocol to functionalize the middle layer of commercial surgical face masks (FMs) with Zn and Cu oxides is proposed in order to obtain antibacterial personal protective equipment. Zinc and copper oxides were synthesized embedded in a polydopamine (PDA) shell as potential antibacterial agents; they were analyzed by XRD and TEM, revealing, in all the cases, the formation of metal oxide nanoparticles (NPs). PDA is a natural polymer appreciated for its simple and rapid synthesis, biocompatibility, and high functionalization; it is used in this work as an organic matrix that, in addition to stabilizing NPs, also acts as a diluent in the functionalization step, decreasing the metal loading on the polypropylene (PP) surface. The functionalized middle layers of the FMs were characterized by SEM, XRD, FTIR, and TXRF and tested in their bacterial-growth-inhibiting effect against *Klebsiella pneumoniae* and *Staphylococcus aureus*. Among all functionalizing agents, Cu_2_O-doped-ZnO NPs enclosed in PDA shell, prepared by an ultrasound-assisted method, showed the best antibacterial effect, even at low metal loading, without changing the hydrophobicity of the FM. This approach offers a sustainable solution by prolonging FM lifespan and reducing material waste.

## 1. Introduction

The COVID-19 outbreak has increased our understanding of how infectious diseases are transmitted and, consequently, the importance of cleaning and disinfecting surfaces [1]. People have learned the importance of using FMs to protect others from exhaled droplets loaded with pathogens. Moreover, during an emergency [2] of such proportions, we were faced with a severe shortage of supplies of devices and materials with antibacterial and antiviral properties. Surgical FMs are the more diffused and cheaper type of mask on the market. This kind of mask is composed by three layers of synthetic fabrics: a PP melt-blown nonwoven layer assembled between two PP spunbonded nonwoven fabrics [3]. The part responsible for filtration is the middle layer of the mask, while the inner part is deputed to absorb moisture, and the outer layer is for repelling the water [4]. FMs can filtrate aerosols and particles larger than 0.7 μm in size [5]. PP makes FMs inexpensive, breathable, water-resistant, and non-toxic [6]. However, the massive use and disposal of plastic personal protective equipment increases plastic pollution [7,8]. Throwing FMs on the ground, the plastic material is exposed to physico-chemical processes in water or soil; after partial degradation, environmental contamination could be due to the release of additives (e.g., stabilizers and plasticizers) present among the FMs’ components [9]. Furthermore the plastic fibers of PP are able to persist for hundreds of years in the environment, giving rise to micro- and nano-plastic pollution [10]; Saliu et al. studied the release of plastic material from FMs in marine water under visible light, concluding that one single FM could release thousands of microscopic fibers [11,12]. The global production of plastic products for the protection against COVID-19 transmission increased a billion times during pandemia [13]; these values indicate the need for urgently finding environmentally sustainable approaches for an ecologically acceptable use of personal protective equipment. This goal could be achieved by combining several strategies, such as encouraging the use of natural plant fibers or other biobased and biodegradable materials in the construction of FMs [14], making higher quality FMs to extend their period of use, and, finally, raising awareness among people about the management of these wastes [15,16]. Until now, the filtration capacity and the strong hydrophobic character of FMs are the only characteristics that determine their effectiveness, while still providing adequate breathability [17]. A method to increase FMs quality is making them more efficient in reducing bacterial contamination by a functionalization with antimicrobial agents. Providing masks with antibacterial properties is a topic much explored recently, as it can prevent the settlement, proliferation, and viability of bacteria. Some works reported the use of Ag [18,19,20], Cu_x_O [21,22], ZnO [23,24] as active functionalizing agents. The inclusion of these metals in personal safety devices may lead to new effective solutions to mitigate the spread of pathogens. The reason for choosing inorganic compounds instead of organic molecules as bacterial-growth-inhibiting agents is due to the huge spectrum of antibacterial activity of the metals: unlike organic molecules, such as polyphenols, metals act by a non-selective mechanism and can be effective even at low load [25]. The antibacterial effect of Zn [26,27] and Cu [21,22] species, and even more of their combination [28], has already been described in the literature, and the mechanism of action has been ascribed to the susceptibility of the cellular membranes and their associated components (e.g., proteins or glycoproteins) to the reactive oxygen species (ROS) produced by metals [29,30]. The Cu and Zn ions toxicity, due even to the ability to penetrate epidermis, is known in the literature [31,32]. The use of metal oxides NPs avoids the metal absorption by the intact skin [33]; nevertheless, the contact with acid fluid, as an acidic sweat, could affect the stability of NPs releasing the corresponding ions [34]. Furthermore, data on the toxicity in animal models of metals and NPs thereof, either through inhalation or ingestion, are available, which suggest the need to consider this issue in terms of the amount of these elements to which the individual is likely to be exposed [35,36].

Methods developed to functionalize FMs involve the use of the following: (i) techniques such as electrospinning [37,38,39], to produce metal-doped fibers for new FMs (functionalization before FMs production); (ii) new more reactive polymers substituting PP, such as poly(ε-caprolactone) [37], polyvinyl alcohol [40], or poly(methylmethacrylate) [39], in order to functionalize FMs after their production in an easier way; and (iii) pretreatments of commercial FMs [17], to make them more reactive to functionalizing methods, like deep coating in antibacterial agent solution [41,42]. Indeed, the low polarity of PP and the lack of functional groups makes the derivatization of already produced FMs difficult. Recently, an activation of the PP layer with acids in hot conditions [17] and a PP pre-treatment with O_2_ plasma to have a PP-O active surface were proposed [43].

In this work, a method to functionalize FMs’ middle layer with metal (Cu, Zn) oxides, through deep coating under heating and in an ultrasound bath without any PP pretreatment, is proposed. The interaction between not-pretreated fibers and metals could be favored by an organic matrix, such as chitosan [23] or dopamine (DA) [44], ensuring the metal adhesion to the fibers without any modification in the PP layer properties [45]. DA is an amino acid present in mussel adhesive proteins where it boasts strong adhesion power [46]; it is used to make the coating on several materials and surfaces [47,48]. DA is able to self-polymerize in an alkaline environment [49,50], through an oxidative polymerization, to form PDA bearing a large number of phenolic, hydroxyl and amino groups [46], which can interact with metals. This polymer boasts a simple and versatile synthesis [51]. Our sustainable protocol involves the presence of PDA acting as shell around the metal NPs used for the FMs’ functionalization. Combining the hydrophobicity of FMs with the antibacterial effect of metal oxides added on FMs’ middle layer, we could improve the FMs protective character and, consequentially, extend their life in terms of efficiency and, therefore, durability, helping to decrease the large amount of used FMs and their impact on environment [52,53].

## 2. Results

### 2.1. Characterization of Functionalizing Agents

The functionalizing agents, in their NP form, were prepared following described procedures, then they were characterized by XRD and TEM.

XRD was used to identify the chemical composition and crystallinity of potential antibacterial agents. The diffraction pattern recorded by XRD for Cu_2_O/PDA (Figure S1a, ESI†) showed peak positions at 2θ values of 29.6°, 36.4°, 42.3°, 61.4°, and 73.5°, corresponding to the (110), (111), (200), (220), and (311) reflection planes of Cu_2_O [54]; all the signals were very sharp due to the crystallinity of the oxide [55].

For sample, for CuO/ZnO, the diffraction peaks at 2θ values 31.8°, 34.5°, 36.3°, 47.6°, 56.6°, 62.9°, 66.5°, 68°, and 69° were assigned to (100), (002), (101), (102), (110), (103), (200), (112), and (201) lattice planes of hexagonal wurtzite structure of ZnO [56,57] that seem unaffected by the presence of the little amount of Cu (Figure S1b, ESI†).

The Cu_2_O/ZnO/PDA diffractogram showed broad peaks attributable to ZnO (2θ values 34°, 36.1°, and 62.5°) [58]; the Cu structure was not identifiable since it was present in a little amount and its signals were hidden by the amorphous PDA and ZnO peaks (Figure S1c, ESI†). To understand the oxidation state of copper oxide, the same synthesis was carried out without the presence of Zn, starting from DA and CuSO_4_·5H_2_O. In fact, the reduction of CuSO_4_ is not affected by the presence of Zn [59] since it is due to the reducing property of PDA; it is known in the literature that PDA can reduce, for example, Ag [60] or Cu [61]. The recorded XRD showed the typical signals of Cu_2_O (broad peaks at 2θ values 37°, 42°, and 61°) [54] (Figure S1g, ESI†).

In the XRD of ZnO (Figure S1d, ESI†), the ZnO species is recognizable from the presence of peaks at 2θ values 31.5°, 34.2°, 36°, 47.3°, 56.4°, 62.6°, and 67.7°, as described above for the sample of CuO/ZnO [56].

The pattern recorded for the Cu_2_O sample (Figure S1e, ESI†) showed the only presence of Cu_2_O, recognizable by the characteristic peaks attributable to Cu_2_O, as in the diffraction pattern of Cu_2_O/PDA [54]. In the sample ZnO/PDA, in which ZnO was synthesized in the presence of PDA, the XRD pattern was disturbed by the amorphous PDA; however, every signal corresponding to ZnO [56] was clearly visible (2θ values 31.7°, 34.5°, 36.1°, 47.6°, 56.5°, 62.6°, and 67.7°) (Figure S1f, ESI†).

**Figure 1:**
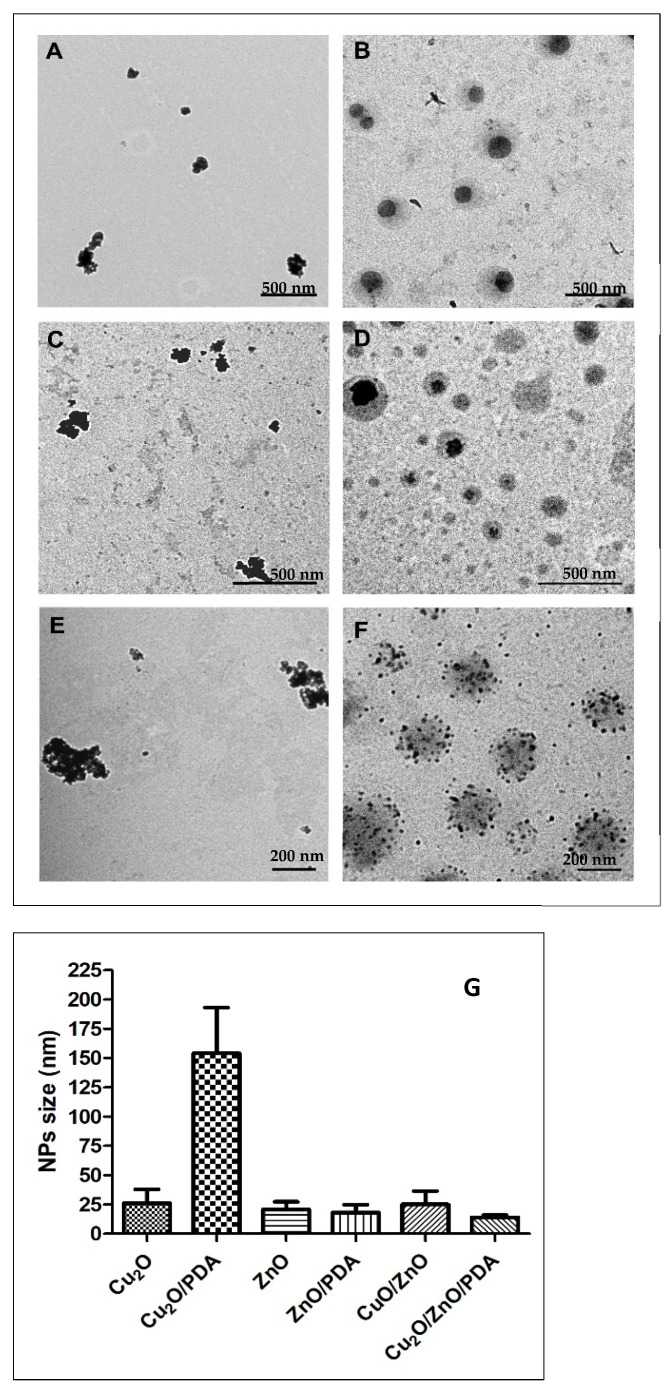
TEM images of (**A**) Cu_2_O, (**B**) Cu_2_O/PDA, (**C**) ZnO, (**D**) ZnO/PDA, (**E**) CuO/ZnO, and (**F**) Cu_2_O/ZnO/PDA. Scale bar 500 nm in (**A**–**D**) and 200 nm in (**E**–**G**). Average diameters of NPs expressed in nm ± SD.

TEM was carried out to evaluate size and shape of the NPs (Figure 1). Cu_2_O NPs were spherical with a diameter of 26 ± 12 nm (Figure 1A). When their synthesis was assisted by PDA, bigger grey spheres were observed with an average diameter of 352 nm, attributable to PDA [62], which contained the spherical Cu_2_O particles with a diameter of 154 ± 39 nm (Figure 1B). CuO/ZnO NPs appeared with spherical shape and dimensions of 25 ± 11 nm (Figure 1E). Cu_2_O/ZnO/PDA NPs showed a homogeneous dispersion of spherical NPs with average diameter of 14 ± 2 nm, mainly enclosed in PDA shells that were 230 nm in size [63,64] (Figure 1F). Even in the case of ZnO, NPs with spherical shape and an average diameter of 20 ± 6 nm were observed (Figure 1C). ZnO prepared in presence of PDA showed again big PDA particles of 144 nm, and spherical ZnO NPs of 18 ± 7 nm; in this case, the size of ZnO NPs was measured for the few single NPs outside the PDA shell, because inside it, the NPs tend to aggregate and it was not possible to distinguish the diameter of each NP (Figure 1D). Comparing all the NPs, Cu_2_O/ZnO/PDA showed the smallest diameter with the most homogeneous distribution in size and, furthermore, their population inside the PDA shell was higher with a minor degree of aggregation (Figure 1F).

### 2.2. Characterization of Functionalized FMs

**Figure 2:**
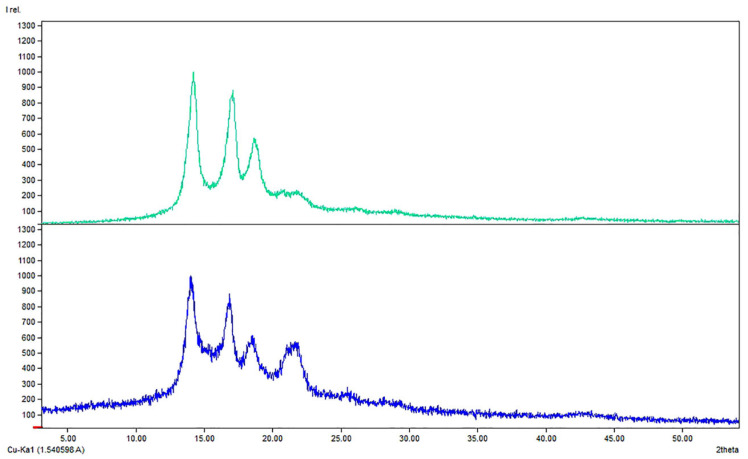
Comparison between XRD patternof (**top**) Blk: PP middle layer of a FM and (**bottom**) sample C: functionalized PP middle layer of a FM with Cu_2_O/ZnO/PDA.

The layers of the functionalized FMs were characterized by XRD, FTIR, SEM, and TXRF. Comparing XRD of FMs and NP-functionalized FMs, the main structure of the polymeric layer seems to be maintained: a typical pattern of PP was observed (COD-inorg 96-155-2372); the peaks of PP were at 2θ values 14.2°, 17°, 18.7°, and 20.7°, due to (110), (045), (130), and (131) planes of the monoclinic α-polypropylene phase (Figure 2) [65,66] After the superficial functionalization occurred on both the faces of FM middle layer, only a decrease of the peaks’ sharpening was appreciable as the deposition of material was made onto the PP surface.

The FTIR spectrum of the non-modified PP middle layer (Figure S2, ESI†) showed main peaks at 2949 and 2866 cm^−1^, corresponding to the asymmetric and symmetric stretching of CH_3_ of the polymer; at 2916 and 2837 cm^−1^, due to asymmetric and symmetric stretching of CH_2_; at 1452 and 1375 cm^−1^, attributable to asymmetric and symmetric bending of CH_3_; at 1166 cm^−1^, corresponding to wagging of CH_3_; at 997 and 840 cm^−1^, for the rocking vibration of CH_3_ and CH_2_; and at 972 cm^−1^, due to C-C stretching [67,68].

Comparing FTIR spectra of FMs before and after the different functionalizations (Figure S2G, ESI†), it is possible to conclude that the main polymeric structure of layer was maintained, as seen in XRD characterization; furthermore, the presence of functionalizing agent was confirmed by the spectra for every sample.

In sample A (Figure S2A, red, ESI†), a broad signal between 1100 and 900 cm^−1^, as in the spectrum of Cu_2_O/PDA (Figure S2A, green, ESI†), and a signal around 630 cm^−1^, all attributable to the stretching of Cu-O bond in Cu_2_O, appeared [69].

In sample B (Figure S2B, red, ESI†), the spectrum showed the signal around 500 cm^−1^, as a confirmation of the presence of species containing metal-oxygen (M-O) bond such as ZnO [70] and CuO [71].

In the FTIR spectrum of sample C (Figure S2C, red, ESI†) the peak present at 1500 can be attributable to NH bending of PDA and that at 1280 cm^−1^ is due to the CH_2_ scissoring of PDA [72].

The FTIR spectrum of sample D (Figure S2D, red, ESI†) showed broad band at 500 cm^−1^, associable to the presence of ZnO NPs [70].

The presence of Cu_2_O in sample E (Figure S2E, red, ESI†) was confirmed by the peaks at 1100 cm^−1^, attributable to the stretching vibration of Cu-O bond in Cu_2_O [69].

The presence of ZnO-PDA in sample F (Figure S2F, red, ESI†) was proved by the little broad band starting from 600 cm^−1^ and attributable to M-O stretching and by the intense peak at 1544 cm^−1^ due to NH bending of PDA; the same signals are [72] present in the spectrum of Sample G (Figure S2G, red, ESI†), in which there is an additional band at 3350 cm^−1^, attributable to OH and NH stretching of the polymer.

**Figure 3:**
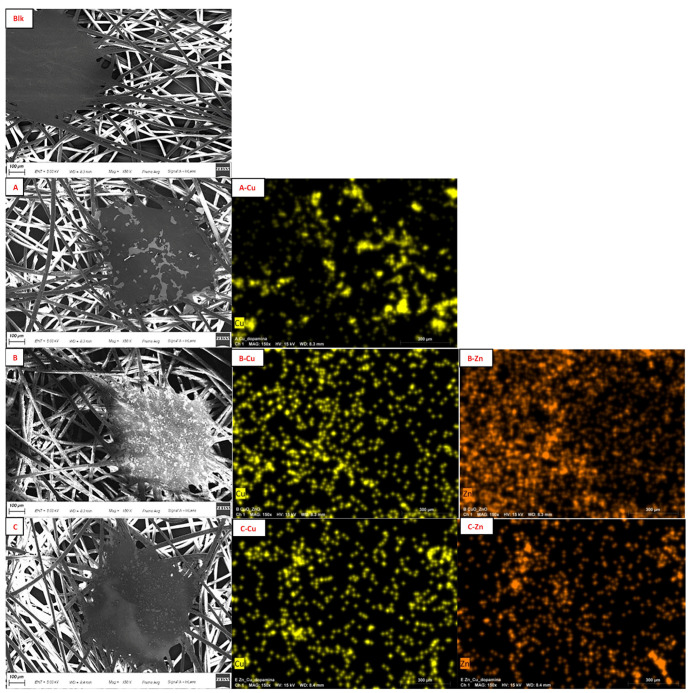

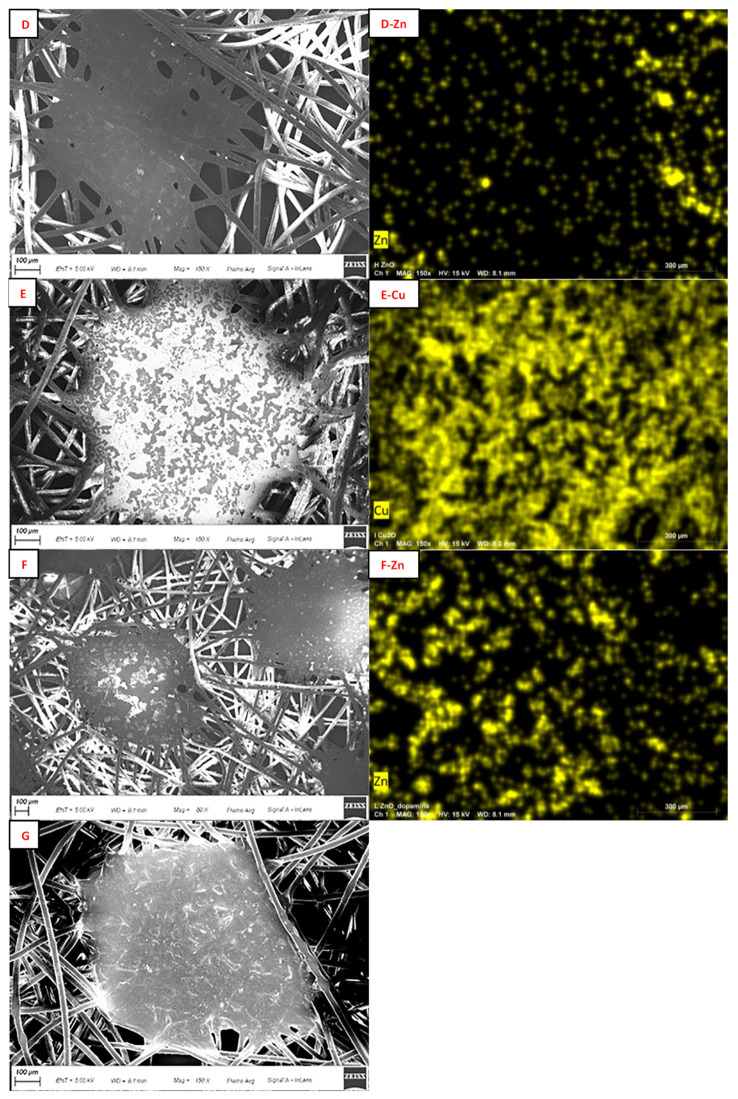
On the left: SEM images of Blk (**Blk**); sample A (**A**); sample B (**B**); sample C (**C**); sample D (**D**); sample E (**E**); sample F (**F**); and sample G (**G**). On the right: elemental mapping of Cu in sample A (**A-Cu**); Cu (**B-Cu**) and Zn (**B-Zn**) in sample B; Cu (**C-Cu**) and Zn (**C-Zn**) in sample C; Zn in sample D (**D-Zn**); Cu in sample E (**E-Cu**); and Zn in sample F (**F-Zn**). Scale bar: 100 μm.

The morphological characteristics of FMs were investigated by SEM (Figure 3). The images showed the fibers with cylindrical shape and smooth surface; at regular distance, there were some more compact islets in which the fibers blended. The analysis confirmed that functionalization occurred in each sample, visible as scales around the fibers or on the islets [73]. Elemental mapping attested the presence of the corresponding metals and showed their distribution on the surface (Figure 3).

Comparing the elemental mapping of the FM functionalized with only Cu (Figure 3(A-Cu),(E-Cu)), it is highlighted that the presence of PDA acted as a diluent and avoided the formation of a continuous layer of Cu_2_O on the PP surface, but the distribution of Cu is not homogeneous in sample A. The same effect was even evident in the comparison of Zn distribution for sample B (without PDA) and C (Figure 3(B-Zn),(C-Zn)); sample C also showed better homogeneous Zn distribution, even comparing with sample F (Figure 3(F-Zn)). In sample G, no visible material on the FM was detected, in fact, the fibers appeared clearer on their surface and some thickenings were observed only around their edges; this evidence attested the capacity of PDA to form coatings and not random spots (Figure 3G) [74]. The EDX results of sample G revealed an increase in the oxygen amount (from 6 to 10% *w*/*w*) and the appearance of a nitrogen signal (Figure S3, ESI†) as an indicator of the presence of PDA.

In Table 1, the percentage (weight of metal per weight of PP layer) of metal supported on the functionalized FM samples were reported. The amount of Zn, revealed by TXRF, in the samples B and D were not so different from each other as well as for sample C and F; presumably NPs size influences the Zn loading since Cu_2_O/ZnO/PDA and ZnO/PDA NPs are bigger than CuO/ZnO and ZnO NPs considering the PDA shell diameters; furthermore, this evidence suggested that the FMs functionalization method is not correlated to the nature of metals but, mainly, to the NPs sizes.

The antimicrobial activity of the FMs was tested against a Gram-positive bacterial species (*Staphylococcus aureus*) and a Gram-negative one (*Klebsiella pneumoniae*). The antibacterial testing method, when applied to the functionalized layer, may result in the inhibition of bacterial growth beneath or in the border proximity of the tissue specimen. Table 2 and Table 3 showed the results of the tests against *S. aureus* and *K. pneumoniae*, respectively, according to the evaluation criteria indicated in the standard ISO 20645 [75,76]. The norm considers two growth inhibitory effects: (i) the absence of growth beneath the specimen, which is more likely associated with an inhibition by contact or proximity and (ii) the formation of a growth inhibition zone around the disc-shaped tissue specimen, where the more the active is diffusing from the tissue the wider the zone of inhibition. The combined effect of these factors determines the score used to classify a tissue material as being antibacterial. Overall, although the inhibition zone diameters produced by the active functionalized FM materials were above the threshold, they were not wide enough to indicate a substantial diffusion, hence a release of NPs from the non-woven tissue.

Materials functionalized with copper species, alone (sample E) or in combination with PDA (sample A), did not demonstrate good effect on *S. aureus* and *K. pneumoniae* growth, as compared to negative controls represented by Blk, Soaked-FM, and FM functionalized with only PDA (sample G). FMs with ZnO alone (sample D) showed an inhibitory activity against *S. aureus* and were of limited efficacy against *K. pneumoniae*; while in presence of PDA (sample F), an opposite behavior was detected. The combination of Cu and Zn species always resulted in a good effect versus both *S. aureus* and *K. pneumoniae* (samples B and C).

## 3. Discussion

All the proposed functionalizing agents were synthesized obtaining NPs with quite small dimensions and spherical shape; only the species prepared in the presence of PDA had an organic shell of bigger size. PDA can produce hybrid coatings [48] promoting interactions with metals [77] and NPs [78], giving an useful contribution to the materials engineering sector [79]. The coating with PDA was selected because of its well-known properties of minimizing any metal leaching [80]. Accordingly, the inhibition zone diameters obtained in our tests indicated that release of NPs from the materials is not high; in fact, PDA increases the adhesiveness between the metal species and the inert PP layer. The catecholamine moiety of PDA is what confers adhesivity, as it is well-known [81]. The other fundamental aspect is the biocompatibility of this polymer, is that it is already used in vivo for the functionalization of NPs, both in the case of hydrophilic and hydrophobic metal surfaces [82]. Thanks to its stability and the ability to modulate its thickness around materials, PDA is widely used [83]; many studies demonstrated the cytocompatibility and negligible cytotoxicity of PDA, even at the nanoscale [84].

**Figure 4:**
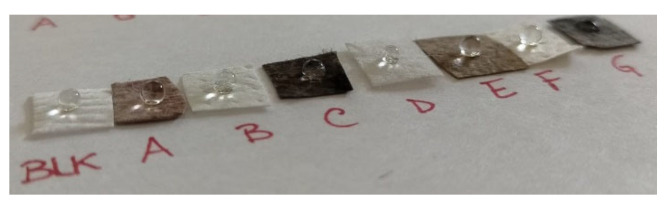
Contact angle of the FM samples, from the left to the right: sample Blk, sample A, sample B, sample C, sample D, sample E, sample F, sample G.

Despite the fact that PDA is more hydrophilic than PP, which could negatively affect the protective capability of the FM [85], the functionalized PP layer is no less hydrophobic than the pristine PP, as can be seen from the contact angle of a droplet of water on the surface of each sample (Figure 4). Only sample G could be considered a hydrophilic material as the droplet doesn’t maintain the spherical shape, increasing its contact surface on the layer, due to the PDA coating and its catecholic moiety [86]. The relative hydrophobic property of metal NPs in PDA shell was also highlighted in a recently published study [87].

In our case, all metal oxide NPs were able to interact with PP leading to FM functionalization, but the protocol involving PDA and metal NPs is convenient because the presence of the organic matrix resulted in a drastic decrease of the total amount of metals in the functionalized FMs. Only in the case of sample A, the amount of Cu_2_O loaded on the FM is comparable to that of sample E, highlighting no effect of PDA presence, most probably because the synthesized Cu_2_O did not show nano size, as their average diameter was more than 100 nm, so the Cu_2_O particles were comparable in size with their PDA shell [88]. Instead, in the case of ZnO NPs, the presence of PDA decreased ZnO loading on the FM from 24% to 3% (Table 1). This is positive, since even with the low Zn amount, the bactericidal effect occurred; so, it is important to use lowest metal content as to have the required antibacterial FMs, avoiding any toxic effect for human skin [89,90]. Cu species used as a functionalizing agent alone did not show antibacterial effect. The low amount of ZnO (when ZnO NPs are embedded in PDA, 3%, Table 1, sample F) functionalizing FMs is enough to make the FM layer antibacterial against *Klebsiella*, but is not quite effective with *Aureus* (Table 2 and Table 3). By addition of a small amount of Cu_2_O, it is possible to generate a synergy between the two metal species [91,92], obtaining great effectiveness even against *S. aureus* (sample C, Table 2) without changing ZnO loading. The increased antimicrobial activity of the CuO-doped ZnO modified-FM is also evident from the good results obtained with sample B against both bacterial species (Table 2 and Table 3). However, compared to the sample C, sample B contains more CuO (0.68% versus 0.18%) and more ZnO (17% versus 3%), since the synthesis of the functionalizing agent was not PDA assisted.

In the literature, the action mechanism of Cu and Zn oxides is well known: they can interact with bacterial cell membranes, leading to their physical disruption through oxidative damage causing by ROS generation [93]; after the loss of cell wall and membranes, a destabilization of normal physiological activities occurs [92,94], such as inhibition of bacterial enzymes [95]. The Cu–Zn synergy could be explained by the improvement of ROS yield at the interface of CuO and ZnO, due to the formation of p-n heterojunctions [96] in the presence of light [97,98], or even in the dark, via a Fenton-type reaction [99].

Hence, Cu_2_O/ZnO/PDA is candidate to be the most appropriate antimicrobial functionalization type. Cu_2_O/ZnO/PDA boasted an easy and fast synthesis thanks to the use of an ultrasound-assisted one-pot synthesis [100,101] that is green, efficient, and cost effective, as it consumes less energy, solvent, and materials [102]. The PDA employment generated an organic shell around the NPs and acted as mild reducing agent for CuSO_4_. Among all samples, Cu_2_O/ZnO/PDA showed the presence of Cu_2_O and ZnO NPs, almost enclosed in the PDA shell, with a good dispersion and characterized by the smallest size compared to the NPs described in this work. Their supporting on FMs did not affect the nature of PP but gave to it an antibacterial property, even at a lower metal amount compared to the functionalization without PDA (sample B). The data in Table 1 demonstrated that the metal loading on FMs was lower for sample C and F because PDA acted as organic filler and led to a lower metal supporting amount.

The available data from the literature may support a discussion on the possible toxicity associated with the use of FM materials functionalizing as copper and zinc oxide NPs [103,104]. It is worth noting that toxicity has been so far evaluated in cell cultures and some animal models. Indeed, the translation of this evidence to humans is not trivial. The weight of the FM middle layer subjected to the functionalization is around 460 mg; so, considering, for example, the sample C designed, synthetized, and tested in this research work as a new active functionalizing agent, it results in 0.8 mg of Cu species and 13 mg of Zn species onto the total surface of a FM (Table 1). Albeit assuming a maximum leaching of 50% it would result in the ingestion or inhalation of 7 mg of metals by the wearer. This quantity is considerably much lower than the levels identified as toxic in animal studies [103,104], also after accounting for the human equivalent dose (HED), which is the presumptive dose that may produce the same toxic effects in humans as the dose that produced toxic effects in animal models.

The proposed functionalization of commercial surgical FMs significantly advances the current state-of-the-art, since, until now, a simple wet method to obtain antibacterial FMs using CuO, ZnO, and PDA together was not reported in the literature. For the first time, PDA was used as organic filler during the functionalization of FMs with inorganic material. The antibacterial agents, employed in published articles, were CuO or ZnO, used alternatively [105]. In presence of only 1.5% of ZnO loaded on FMs, it was reported an intense decrease of the bacteria growth [106], but to break down it, a higher supported metal amount [107,108,109] was required. The proposed combination of copper and zinc oxides gives rise to synergy and so leading to 99% bacterial inhibition, even at a very low metal amount.

## 4. Materials and Methods

### 4.1. Materials

Sodium lignosulphonate was supplied for free by Burgo Group S.p.A. (Tolmezzo, Italy); ethyl alcohol was purchased from Carlo Erba Reagents. All the other chemicals were purchased from Merck and used without any purification.

The micro-organisms employed for antibacterial studies were gram-positive bacteria (*S. aureus*) and gram-negative bacteria (*K. pneumoniae*); in all experiments, the growth medium used was the Nutrient Broth Oxoid (Fisher Scientific, Segrate, Italy).

Non-woven multi-layered FMs (Medea, Italy) were purchased from a local supermarket. The only FM portion taken into consideration is the melt-blown nonwoven middle layer that is primarily responsible for air filtration.

### 4.2. Instruments

The ultrasonic bath was an Elmasonic select 60 (frequency 37 kHz), set to pulse mode. The sonicator with probe was Cole Parmer Ultrasonic Processor GEX 400 set at 80% amplitude.

XRD analysis was performed on a Miniflex II Rigaku automated power XRD system (Cu Kα radiation, 45 kV, 100 mA) (RINT2500 diffractometer of RIGAKU Co, Tokyo, Japan). Diffraction data were recorded using continuous scanning at 3° min^−1^, with 0.010° steps. To analyze the functionalizing agents, they were dried and ground in a mortar to obtain a powder, only Cu_2_O/ZnO/PDA was previously washed with water since the presence of salts arising from synthesis hid the sample XRD profile. FMs samples were put on the slide with double sided adhesive tape.

FTIR analysis was carried out with the Fourier Transform Infrared Spectrophotometer Shimadzu IRAffinity-1S. The functionalizing agents were analyzed as dried powder and FMs without any treatment.

TEM analyses were performed with a 120 kV JEM-1400 Flash Transmission Electron Microscope (Jeol Ltd., Tokyo, Japan) equipped with a CMOS camera Matataki and TEM Center software (Jeol Ltd., Tokyo, Japan). The samples were prepared using 5 mL of a solution of 10 mg functionalized agent in 30 mL ultrapure water; the solution was sonicated in an ultrasonic bath for 3 min, then it was deposited onto 3 mm carbon films on 300 mesh grids made of copper (Agar Scientific Ltd., Stansted, UK), and the solvent was left to evaporate few hours at room temperature. The NPs size and shape were determined from electron micrographs of nonoverlapping regions randomly collected using the TEM Center Software (SightX Viewer Ver.2.1.23.1656).

SEM images were acquired by scanning electron microscopy using a Zeiss Sigma 300 FE equipped with a Bruker Quanta EDX detector. FM sections were put on aluminum stubs and they were attached with double-sided conductive carbon pads; over them, a conductive chromium film was deposited by a metallizer Quorum mod. Q150T-ES.

A Total Reflection X-Ray Fluorescence Spectrometer (TXRF), used to quantify the metals amount (Zn and Cu) on functionalized FMs, was a Horizon TXRF benchtop spectrometer (GNR, Agrate Conturbia (NO), Italy) with a source X-ray generator LFF Mo/W (max output voltage 40 kV, max output current 15 mA) with a multilayer monochromator of W/Si and the excitation energy Mo Kα of 17.44 keV; the detector type was a Ketech SDD, with a 1 mm graphene window, window detector active area of 40 mm^2^, and measurement time/sample of 600 s. The internal standard used was Co, which was added to each sample to obtain intensity signals comparable with the analytes. FMs were put into a PFA bottle (Savillex, Eden Prairie, MN, USA), weighed, and with Co as internal standard. The samples were treated with 10% HNO_3_ acid solution in ultrapure water (8 mL) at 140 °C for 1.5 h. A total of 10 mL was sampled from the solution to a pre-siliconized quartz reflector, which was then dried at 130 °C on a hot-plate.

To detect the contact angle of a water droplet on the FMs, 10 mL of deionized water was deposited on each FM sample.

### 4.3. Syntheses of Functionalizing Agents

Cu_2_O/PDA: 0.468 g of CuSO_4_·5H_2_O and 0.354 g of dopamine hydrochloride were dissolved in 2.5 mL of water, then 12 mL of aqueous 5 M NaOH was added into the solution after 15 min; the mixture was kept at 100 °C for 24 h. The precipitate was centrifugated at 3000 rpm, washed with water and ethyl alcohol, and dried at 80 °C.

CuO/ZnO: ZnO was prepared by a modified protocol using sodium lignosulphonate [110,111]: 1 g of Zn(CH_3_COO)_2_·2H_2_O, 0.8 g of sodium lignosulphonate, and 0.36 g of NaOH were dissolved in 50 mL of ethyl alcohol; the solution was left at 80 °C for 12 h; the obtained precipitate was filtered, washed with water and ethyl alcohol, and finally calcined at 700 °C for 4 h in air. A portion of synthesized ZnO particles (0.1 g) was dispersed in 20 mL of water and 0.012 g of CuSO_4_·5H_2_O was added; the solution was kept at 100 °C for 12 h, and finally, the precipitate was filtered and washed with water and ethyl alcohol and then calcined at 400 °C for 4 h in air [56].

Cu_2_O/ZnO/PDA: 0.32 g of dopamine hydrochloride was dissolved in 20 mL of water and sonicated with the probe sonicator for 5 min. Then, 0.4 g of Zn(CH_3_COO)_2_·2H_2_O and 0.03 g of CuSO_4_·5H_2_O, dissolved in 20 mL of water, were added to the DA solution; the mixture was sonicated for 5 min. Finally, pH was adjusted at 8 with NaOH solution and then it was sonicated for further 5 min while cooling the container with ice. The obtained dark solution was used to functionalize the FMs [28,112].

ZnO NPs: 1.48 g of Zn(CH_3_COO)_2_·2H_2_O was dissolved in 50 mL of ethyl alcohol at 60 °C. Next, 30 mL of ethyl alcohol containing 0.76 g of KOH was added drop-by-drop to the previous solution; then, it was left at 60 °C for 3 h under magnetic stirring. The obtained white precipitate was centrifugated at 3000 rpm, washed with water and ethyl alcohol, and finally dried at 80 °C for 20 min [113].

Cu_2_O NPs: 0.468 g of CuSO_4_·5H_2_O were dissolved in 2.5 mL water. Then, 12 mL of NaOH 5 M was added, and after 0.371 g of sodium ascorbate was added, the solution was kept for 24 h at 100 °C. The formed precipitate was centrifugated at 3000 rpm, washed with water, and dried at 80 °C for 20 min [114].

ZnO/PDA: 1.28 g of dopamine hydrochloride and 1.48 g of Zn(CH_3_COO)_2_·2H_2_O were dissolved in 30 mL of water and stirred for 10 min at 60 °C; later, 10 mL of NaOH 2 M aqueous solution were added and the mixture was stirred for 12 h at 100 °C. The obtained precipitate was washed with water and ethyl alcohol and dried at 80 °C for 30 min.

PDA: 0.3 g of dopamine hydrochloride was dissolved in 20 mL of water and the pH was increased up to 12 by addition of 2 M NaOH. The mixture was left at room temperature for 2 h in order to allow the polymerization of DA. The obtained solution was used to functionalize the FMs.

### 4.4. FMs Functionalization Procedure

**Figure 5:**
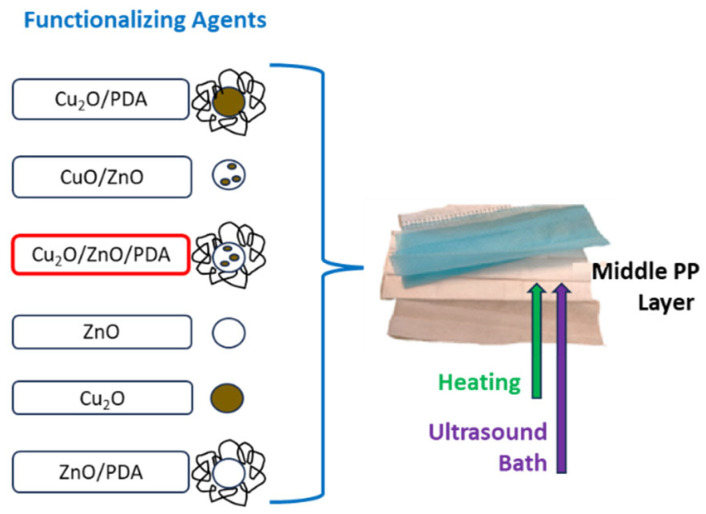
Schematic representation of FM middle-layer functionalization with different synthetized antibacterial agents.

The functionalization of FMs was carried out by dipping the middle layer of the FMs in 12 mL of solution (8 mL water + 4 mL ethyl alcohol) containing 200 mg of the functionalizing agent. The FMs in the alcohol/water mixture were sonicated in a bath for 2 h, then the mixture was heated at 100 °C for 12 h (Figure 5); after this, the FMs were collected and left to cure for 24 h; finally, they were washed with water and ethyl alcohol and dried at room temperature. A FM portion underwent the same procedure of deep coating, in water/ethyl alcohol solution without any functionalizing agent under ultrasound and thermal treatments, to obtain a comparison sample (Soaked-FM). All abbreviations used for the FMs are reported below:

Sample Blk: not-functionalized FM

Sample Soaked-FM: treated FM in water/ethyl alcohol solution

Sample A: functionalized FM with Cu_2_O/PDA

Sample B: functionalized FM with CuO/ZnO

Sample C: functionalized FM with Cu_2_O/ZnO/PDA

Sample D: functionalized FM with ZnO

Sample E: functionalized FM with Cu_2_O

Sample F: functionalized FM with ZnO/PDA

Sample G: functionalized FM with PDA

### 4.5. Antibacterial Activity Tests

The tests were carried out following a protocol based on that described in the standard ISO 20645 [75]. The rationale of this test is to assess the antibacterial activity of a fabric by contact of the material with a lawn of bacteria. The activity was evaluated against *Staphylococcus aureus* (strain ATCC 6538P) and *Klebsiella pneumoniae* (strain ATCC 4352). The sole modification to the ISO protocol was that the tissue samples derivatized with the different types of treatments were cut with a borer producing discs with a diameter of 1.3 cm instead of the indicated 2.5 cm. The test results were expressed according to the evaluation guidelines defined in the standard, i.e., by measuring the growth inhibition zone (H) around the specimen calculated according to the formula H = (D − d)/2 where D and d indicated, respectively, the total diameter (sample + growth inhibition zone) and the sample diameter, both expressed in mm. The final values resulted from the average of four measurements. Additionally, the reduction or absence of growth beneath the disc was qualitatively assessed as suggested by the ISO standard. The last evaluation was also assisted by light microscopy.

## 5. Conclusions

The recurrent pandemics and epidemics caused by respiratory pathogens highlight our society’s unpreparedness to deal with them. The prolonged survival of pathogenic microorganisms in the environment, including on surfaces and in aerosols, has contributed to the observed increase in infection rates. Here, oxides of Cu and Zn, as NPs, embedded in a PDA shell, were employed to functionalize FMs to make them antibacterial. PDA was involved to create a homogeneous film onto the FMs’ surface as a compatibilizer because it is able to interact with surfaces, and, also, due to its catechol, hydroxyl, and amine groups, can itself be a scaffold for further functionalization [43,115]. Hence, PDA could be defined as a sort of “diluting” agent, since when present, it led to a lower net metal loading, obtaining likewise an antibacterial effect on the FMs.

FMs functionalized by Cu_2_O/ZnO/PDA (sample C) showed the best anti-*S. aureus* and anti-*K. pneumoniae* activity, due to the synergic effect of the Cu_2_O/ZnO combination, minimizing, at the same time, the relative metal amounts thanks to the presence of PDA.

In this work, only the intermediate layer of the FM was functionalized to avoid direct contact between metals and skin, since a third layer separates the metal oxide NPs from the wearer’s face. Furthermore, this last layer is highly hydrophobic, so it does not allow impregnation of the inner part with fluids, such as saliva or sweat.

A novel cheap and fast method to transform already commercialized FMs is proposed without changing in the polymeric main structure of non-woven fabric, as observed by XRD and FTIR. The combination of many convenient aspects, like the use of cheap metals, such as Cu and Zn, in their non-toxic oxide form, the employment of a green and multifunctional PDA coating, and the choice of simple methodologies for the functionalization step, make this antibacterial FM production a new sustainable approach to increase the half-life of FMs and decrease the amount of wasted material.

## Figures and Tables

**Table 1:** TXRF data of the FMs.

Sample	Components	Cu (% *w*/*w*)	Zn (% *w*/*w*)
A	Cu_2_O/PDA	20.57	0.00
B	CuO/ZnO	0.68	17.20
C	Cu_2_O/ZnO/PDA	0.18	2.86
D	ZnO	0.00	23.80
E	Cu_2_O	19.70	0.00
F	ZnO/PDA	0.00	2.60
Blk	---	0.00	0.00

**Table 2:** Anti-*Staphylococcus aureus* activity of different FMs samples.

Sample	Plate–Top View with Disc	Plate–Bottom View with Disc	Inhibition Zone (mm)	Plate–Top View without Disc	Growth	Final Assessment
BlK	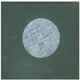	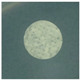	0	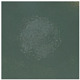	Heavy	Insufficient effect
Soaked-FM	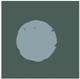	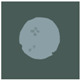	0	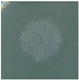	Heavy	Insufficient effect
A	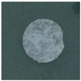	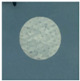	0	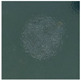	Heavy	Insufficient effect
B	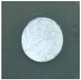	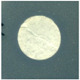	>1	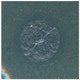	None	Good
C	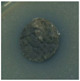	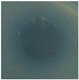	>1	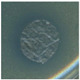	None	Good
D	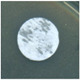	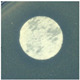	>1	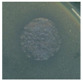	None	Good
E	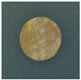	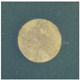	0–1	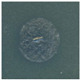	Slight to moderate	Limited efficacy
F	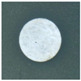	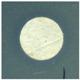	0–1	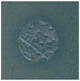	Slight to moderate	Limited efficacy
G	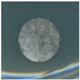	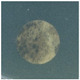	0	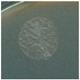	Heavy	Insufficient effect

**Table 3:** Anti-*Klebsiella pneumoniae* activity of different FMs samples.

Sample	Plate–Top View with Disc	Plate–Bottom View with Disc	Inhibition Zone (mm)	Plate–Top View without Disc	Growth	Final Assessment
Blk	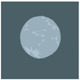	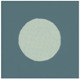	0	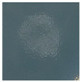	Heavy	Insufficient effect
Soaked-FM	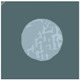	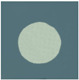	0	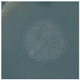	Heavy	Insufficient effect
A	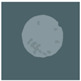	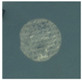	0	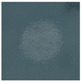	Heavy	Insufficient effect
B	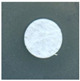	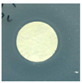	>1	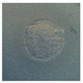	None	Good
C	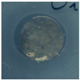	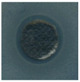	>1	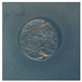	None	Good
D	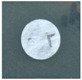	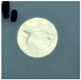	0–1	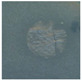	Slight to moderate	Limited efficacy
E	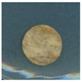	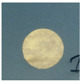	Variable *	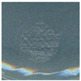	Variable *	Unclassified *
F	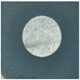	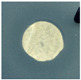	>1	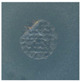	None	Good
G	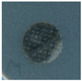	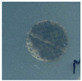	0	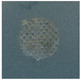	Heavy	Insufficient effect

* Variable response. Activity could not be classified according to the evaluation criteria.

## Data Availability

Data available on request due to restrictions. The data presented in this study are available on request from the corresponding author.

## Supplementary Materials

The following supporting information can be downloaded at: https://www.mdpi.com/article/10.3390/molecules29184512/s1, Figure S1: XRD patternof Cu_2_O/PDA, CuO/ZnO, Cu_2_O/ZnO/PDA, ZnO, Cu_2_O, ZnO/PDA, and Cu_2_O/PDA by ultrasonic-assisted synthesis; Figure S2: FT-IR spectra of Sample A, Sample B, Sample C, Sample D, Sample E, Sample F, and Sample G; Figure S3: EDX analysis of non-treated FMs and treated FMs with PDA; Figure S4: EDX analysis of Sample A, Sample B, Sample C, Sample D, Sample E, and Sample F.
