# Supplementary material for: Cu-ZnO Embedded in a Polydopamine Shell for the Generation of Antibacterial Surgical Face Masks
Source: Molecules. 2024 Sep 23;29(18):4512. doi: 10.3390/molecules29184512 (PMC11434467; doi:10.3390/molecules29184512)
Supplement: Supplementary file 1 [file molecules-29-04512-s001.zip › molecules-3165198-supplementary.pdf]

Article

Cu-ZnO Embedded in a Polydopamine Shell for the Generation of Antibacterial Surgical Face Masks

Nicola d'Alessandro [1,2,3], Francesca Coccia [4,*], Luca Agostino Vitali [5], Giorgia Rastelli [6], Amedeo Cinosi [7], Andrea Mascitti [1] and Lucia Tonucci [2,4]

[1] Department of Engineering and Geology, "G. d'Annunzio" University of Chieti-Pescara, Viale Pindaro 42, 65127 Pescara, Italy; nicola.dalessandro@unich.it (N.d.); andrea.mascitti@alumni.unich.it (A.M.)

[2] TEMA Research Center, University "G. d'Annunzio" of Chieti-Pescara, 66100 Chieti, Italy; lucia.tonucci@unich.it

[3] UdA-TechLab Research Center, University "G. d'Annunzio" of Chieti-Pescara, 66100 Chieti, Italy

[4] Department of Socio-Economic, Managerial and Statistical Studies, "G. d'Annunzio" University of Chieti-Pescara, Via dei Vestini, 31, 66100 Chieti, Italy

[5] School of Pharmacy, University of Camerino via Gentile III da Varano, 62032 Camerino, Italy; luca.vitali@unicam.it

[6] Department of Neuroscience, Imaging and Clinical Science, "G. d'Annunzio" University of Chieti-Pescara, Via dei Vestini, 31, 66100 Chieti, Italy; giorgia.rastelli@unich.it

[7] G.N.R. s.r.l., via Torino 7, 28010 Agrate Conturbia, Italy; info@gnr.it

* Correspondence: francesca.coccia@unich.it

INDEX

XRD ANALYSIS 2

FT-IR ANALYSIS 3

EDX ANALYSIS 4

XRD ANALYSIS

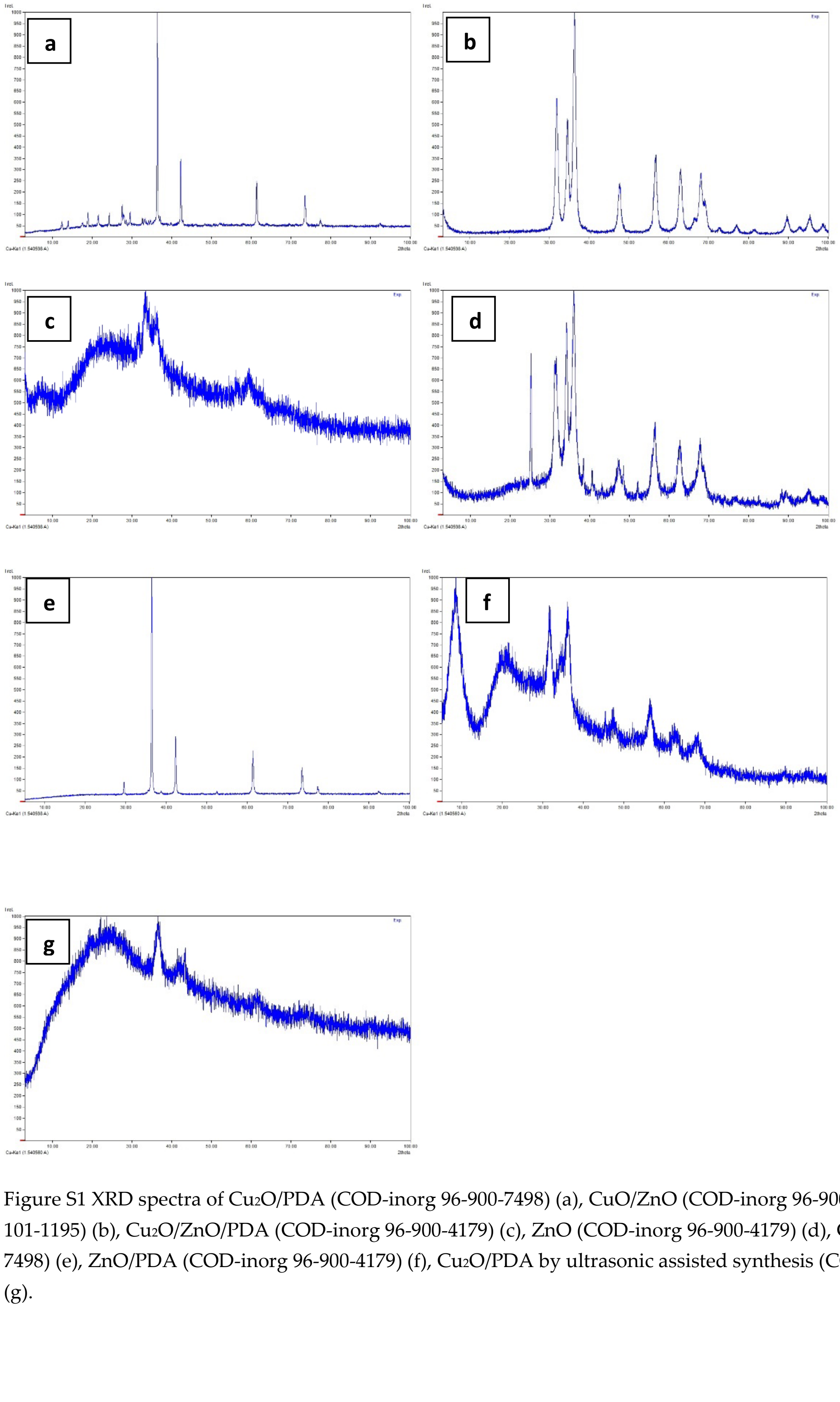

Figure S1 XRD spectra of Cu_2O/PDA (COD-inorg 96-900-7498) (a), CuO/ZnO (COD-inorg 96-900-4179; COD-inorg 96-101-1195) (b), Cu_2O/ZnO/PDA (COD-inorg 96-900-4179) (c), ZnO (COD-inorg 96-900-4179) (d), Cu_2O (COD-inorg 96-900-7498) (e), ZnO/PDA (COD-inorg 96-900-4179) (f), Cu_2O/PDA by ultrasonic assisted synthesis (COD-inorg 96-900-7498) (g).

FT-IR ANALYSIS

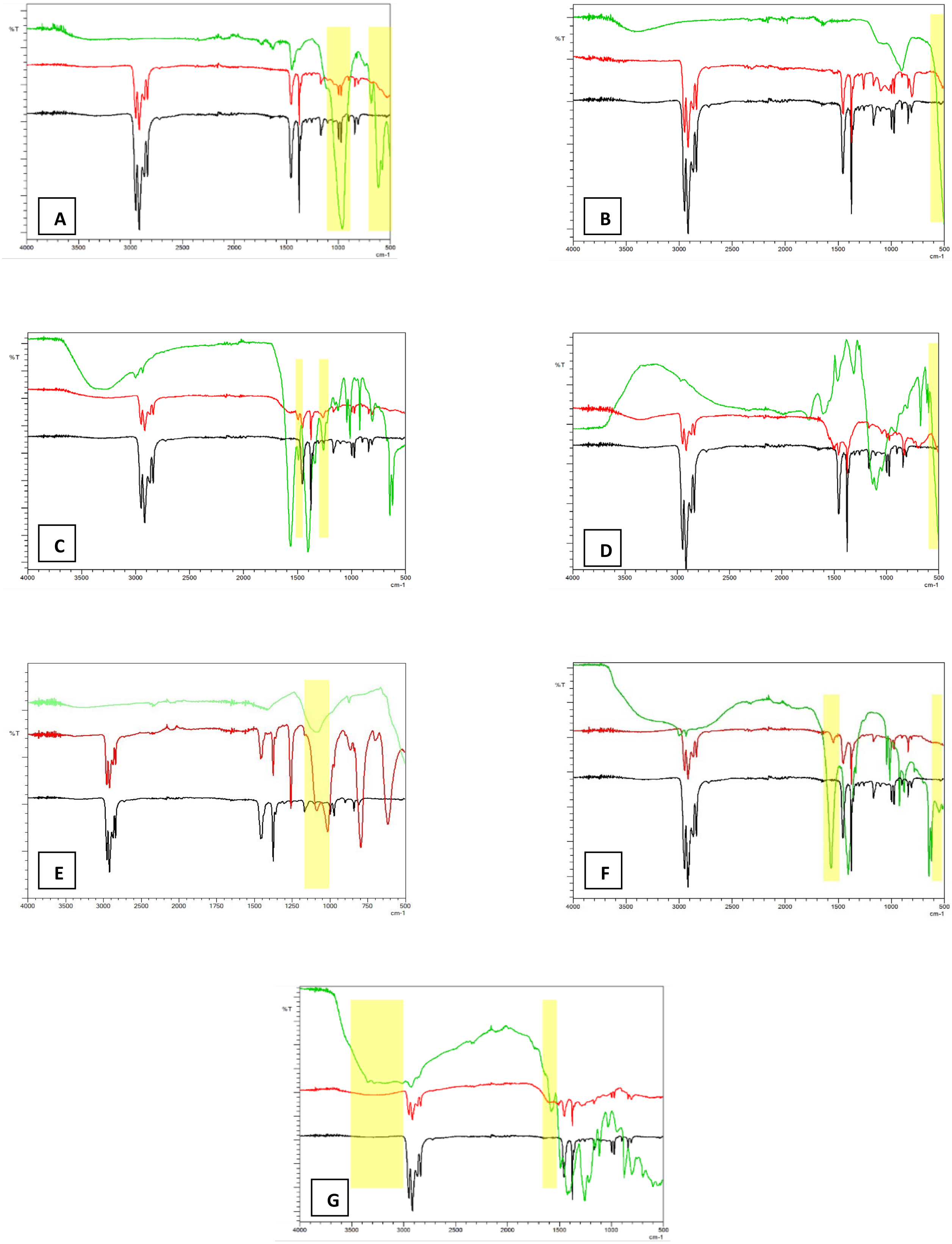

Figure S2 FT-IR spectra of Sample A (A), Sample B (B), Sample C (C), Sample D (D), Sample E (E), Sample F (F), Sample G (G).

EDX ANALYSIS

Element	Norm. Mass (%)	Rel. error (%)
**C**	93.9	10.8
**O**	6.1	16.7

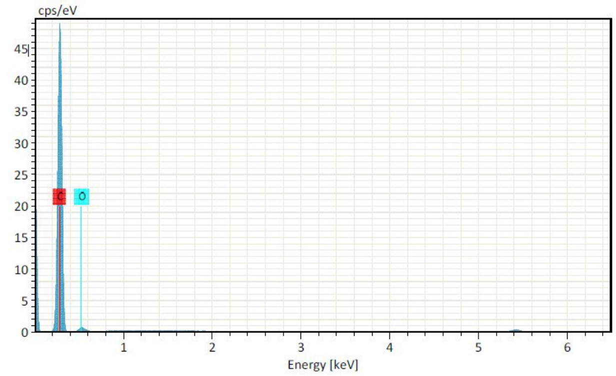

Element	Norm. Mass (%)	Rel. error (%)
**C**	84.5	11.2
**N**	2	34.7
**O**	11	16
**Si**	2.5	5.4

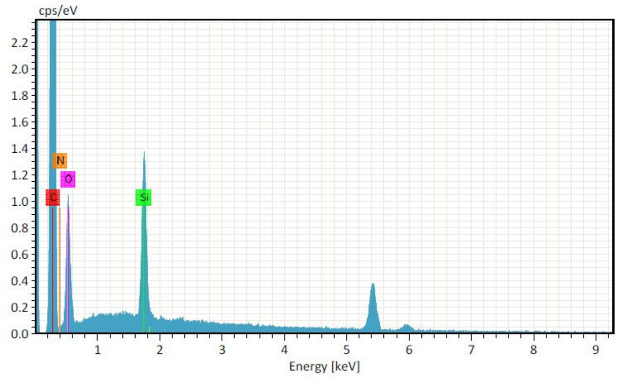

Figure S3 EDX analysis of (up) non treated FM and (down) treated FM with PDA (Sample G)

Element	Norm. Mass (%)	Rel. error (%)
**C**	82.4	11.2
**Cu**	7.1	4.2
**O**	9.8	15.8
**Si**	0.3	13.8

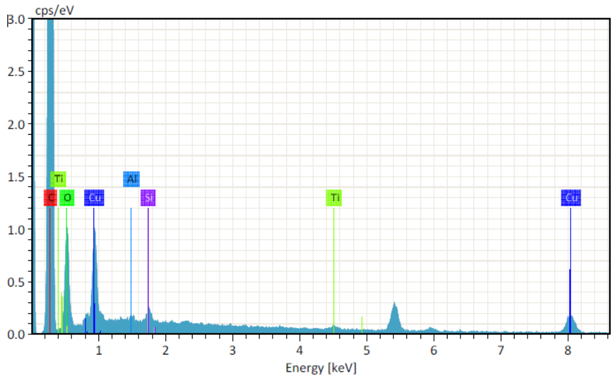

Element	Norm. Mass (%)	Rel. error (%)
**C**	72.3	11.4
**Cu**	0.8	10.9
**O**	12.0	14.6
**Zn**	14.1	4.0

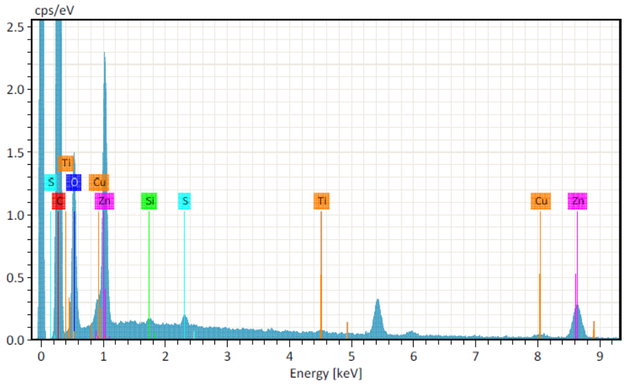

Element	Norm. Mass (%)	Rel. error (%)
**C**	84.9	11.2
**Cu**	1.0	9.6
**O**	9.7	16.4
**Zn**	2.7	6.6

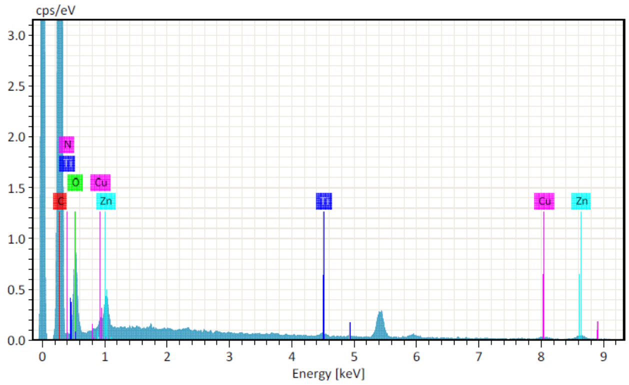

Element	Norm. Mass (%)	Rel. error (%)
**C**	87.8	11.1
**Zn**	2.8	6.4
**O**	7.3	17.2
**Si**	0.2	20.3

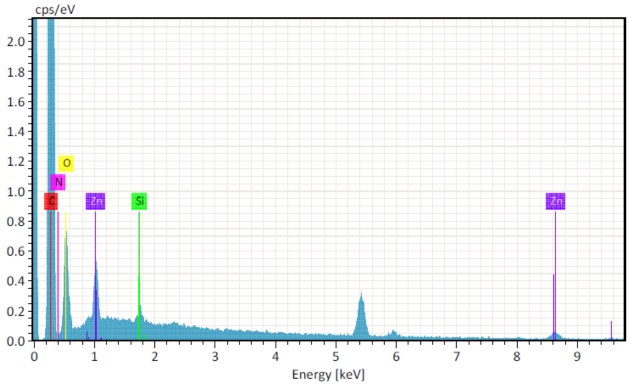

Element	Norm. Mass (%)	Rel. error (%)
**C**	50.2	12.0
**Cu**	41.2	3.4
**O**	5.4	16.3
**Si**	3.0	6.4

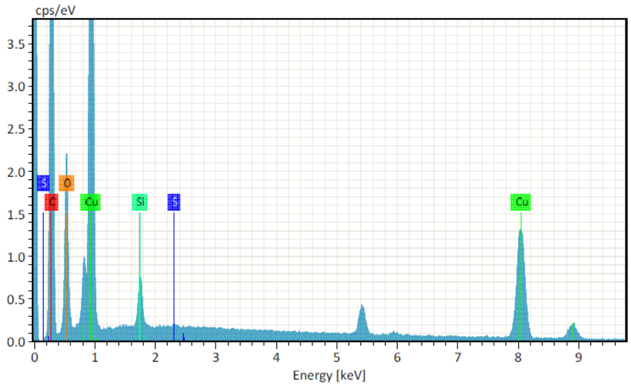

Element	Norm. Mass (%)	Rel. error (%)
**C**	74.0	11.3
**Zn**	6.1	4.6
**O**	17.1	13.8
**Si**	3.0	5.3

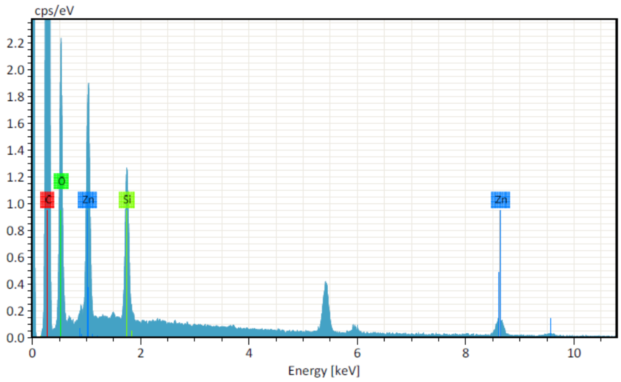

Figure S4 From the top to the bottom: EDX analysis of Sample A, Sample B, Sample C, Sample D, Sample E, Sample F.
